# Expression Profiling Analysis Reveals Key MicroRNA–mRNA Interactions in Early Retinal Degeneration in Retinitis Pigmentosa

**DOI:** 10.1167/iovs.18-24091

**Published:** 2018-05

**Authors:** Ander Anasagasti, Maitane Ezquerra-Inchausti, Olatz Barandika, Maider Muñoz-Culla, María M. Caffarel, David Otaegui, Adolfo López de Munain, Javier Ruiz-Ederra

**Affiliations:** 1 Neuroscience Area, Sensorial Neurodegeneration Group, Biodonostia Health Research Institute, San Sebastian, Spain; 2 RETICS OFTARED, National Institute of Health Carlos III, Ministry of Economy and Competitiveness, Spain; 3 Neuroscience Area, Multiple Sclerosis Group, Biodonostia Health Research Institute, San Sebastian, Spain; 4 Spanish Network on Multiple Sclerosis (Red Española de Esclerosis Múltiple); 5 Oncology Area, Biodonostia Health Research Institute, San Sebastian, Spain; 6 Ikerbasque, Basque Foundation for Science, Bilbao, Spain; 7 Department of Neurology, Donostia University Hospital, San Sebastian, Spain; 8 Centro de Investigaciones Biomédicas en Red Sobre Enfermedades Neurodegenerativas, Instituto Carlos III, Ministerio de Economía y Competitividad, Spain; 9 Department of Neuroscience, University of the Basque Country, San Sebastian, Spain

**Keywords:** retinitis pigmentosa, miRNA, rd10, network analysis, miRNA–mRNA interaction

## Abstract

**Purpose:**

The aim of this study was to identify differentially expressed microRNAs (miRNAs) that might play an important role in the etiology of retinal degeneration in a genetic mouse model of retinitis pigmentosa (rd10 mice) at initial stages of the disease.

**Methods:**

miRNAs–mRNA interaction networks were generated for analysis of biological pathways involved in retinal degeneration.

**Results:**

Of more than 1900 miRNAs analyzed, we selected 19 miRNAs on the basis of (1) a significant differential expression in rd10 retinas compared with control samples and (2) an inverse expression relationship with predicted mRNA targets involved in biological pathways relevant to retinal biology and/or degeneration. Seven of the selected miRNAs have been associated with retinal dystrophies, whereas, to our knowledge, nine have not been previously linked to any disease.

**Conclusions:**

This study contributes to our understanding of the etiology and progression of retinal degeneration.

Retinitis pigmentosa (RP) is a heterogeneous collection of inherited retinal degeneration diseases that leads to vision impairment and for which there is no standard treatment. It is characterized by the progressive death of retinal photoreceptor cells. The primary defect underlying RP affects the function of photoreceptors and/or retinal pigment epithelium cells, where molecular and cellular mechanisms trigger the apoptotic degeneration of rods and in many cases cones.

Despite there being more than 350 genes and/or loci associated with retinal dystrophies, the precise mechanisms that link mutations in these genes with retinal degeneration are still not well defined. In addition to many molecular pathways involved in both survival and death of photoreceptors and RPE cells, there is growing evidence of the role of epigenetic mechanisms as key players in gene expression regulation, retinal development and function, and photoreceptors survival.^1,2^ These include effects mediated by microRNAs (miRNAs), small RNAs that partially inhibit the translation of a transcript or cause mRNA degradation.^3^ According to the current version of the miRBase database (www.mirbase.org), more than 2000 human miRNAs have been identified, each of which targets several hundreds of different mRNAs, mostly due to the fact that most target sites on the mRNA have only partial base complementarity with their corresponding miRNA. Moreover, individual mRNAs may contain multiple binding sites for different miRNAs, resulting in a complex regulatory network. Taken together, it appears that relatively few miRNAs can regulate as many as 50% to 60% of genes in humans and other mammals.^4^

Control of protein expression mediated by miRNA modulation has been described as a widely used mechanism for posttranscriptional regulation of metabolic pathways.^5,6^ There is growing evidence of the involvement of miRNAs dysregulation in a broad spectrum of health problems, including various types of cancer and heart and inflammatory diseases, as well as in hereditary diseases such as cystic fibrosis.^7–15^

Current molecular tools have allowed the selective modulation of miRNA expression. This has led to the development of what will become the first clinical trials based on an antisense approach seeking to regulate miRNA function in patients with cardiovascular conditions.^16^

More than 250 miRNAs have been identified in the retina, and recent studies have shown the relevance of Dicer, a key enzyme in miRNA biogenesis, in photoreceptor survival.^2,17^ Similar to many other human conditions, retinal diseases also involve miRNA dysregulation. Indeed, there is a growing list of miRNAs that are known to be differentially expressed in retinal disorders in humans and in animal models, including miR-9, miR-34a, miR-125b, and miR-155 in macular degeneration,^18,19^ miR-146a and miR-195 in diabetic retinopathy,^20,21^ and miR-125a and miR-17 in retinoblastoma.^22,23^ In addition, a group of antiapoptotic miRNAs (miR-155, miR-146a, and miR-29b) was found to be differentially expressed during the chronic cell death stage in canine models of retinal degeneration.^24^ Furthermore, a common pattern of aberrantly expressed miRNAs was described in four murine models of RP where miR-1, miR-133, and miR-142 were upregulated despite different genes (*Rho* and *Rds*) and inheritance patterns being involved.^25,26^

To identify potential miRNAs associated with the etiology of RP, we used the retinal degeneration 10 (rd10) mouse: a well-established mouse model of autosomal recessive RP, which carries a spontaneous point mutation in exon 13 of the β subunit of the rod phosphodiesterase gene (*Pde6β*). This mutation results in deregulation of intracellular calcium homeostasis that leads to photoreceptor cell death via apoptosis.^27–29^ Mutations in the *PDE6β* gene also cause RP in humans accounting for approximately 5% of all autosomal dominant RP cases worldwide.^30,31^ Therefore, rd10 mice provide a useful and widely used model of RP that closely resembles the human disease emulating the progression of typical autosomal recessive RP.

In summary, we analyzed the expression pattern of miRNAs and associated mRNAs during the early stages of retinal degeneration. Our results provide valuable insights into the molecular mechanisms underlying RP, including the identification of differentially expressed miRNAs, and the association of predicted mRNA targets in the retina of the rd10 mice. These include both miRNAs previously reported in retinal studies, such as miR-1a, miR-133a, miR-146a, miR-17, miR-29b, and miR-155, and miRNAs not previously ascribed to any disease, namely miR-6937, miR-6240, miR-3473b, and miR-6970. This study might also help to identify potential miR-based therapeutic approaches.

## Materials and Methods

### Ethics Statement and Animal Handling

Animal handling and experiments were conducted in accordance with the ARVO Statement for the Use of Animals in Ophthalmic and Vision Research and were approved by the Animal Care and Use Committee of Donostia University Hospital and the Clinical Research Ethics Committee of the Basque Country, Spain. Studies were performed using age-matched wild-type (WT) C57BL/6J mice as controls and a congenic inbred strain of rd10 mice, both purchased from the Jackson Laboratory (Bar Harbor, ME, USA). Animals were maintained under a 12-hour light/12-hour dark cycle at 22°C, with controlled humidity, ranging between 45% and 55%, and with water and food provided ad libitum at the Animal Facility of BioDonostia Health Research Institute in San Sebastian (Spain).

### Sample Collection

Rd10 and age-matched WT mice were euthanized by cervical dislocation following CO_2_ inhalation. All mice were euthanized at the same time of day to minimize possible bias from circadian effects on miRNA expression.

Retinal tissue from each animal was isolated and kept at 4°C within a few minutes until use. Total RNA isolation was performed using the miRNeasy Mini Kit and RNase-Free DNase Set (Qiagen, Woburn, MA, USA), in accordance with the manufacturer's protocol. RNA was stored at −80°C until use. Only those RNA samples with A260/A280 ratios greater than 1.8 were used, as determined by a Nano-Drop ND-1000 spectrophotometer (Nanodrop Technologies, Wilmigton, DE, USA). Furthermore, sample quality was tested in each of the different RNA expression profiling arrays described below.

Eyes were enucleated, fixed in PBS containing 4% paraformaldehyde, and cryoprotected in PBS containing 30% sucrose. They were then embedded in OCT (Tissue Tek, Sakura Finetek, Tokyo, Japan) and sectioned at 7 μm in a cryostat.

### Time Course of Photoreceptor Cell Death

We used a terminal deoxynucleotidyl transferase-mediated dUTP nick-end labeling (TUNEL) assay to study the photoreceptor cell death process to select the time points for collection of retinal samples for our miRNA/mRNA expression studies. We analyzed retinal sections from postnatal days 13 to 22 (P13 to P22), obtained at 12-hour intervals using the DeadEnd Fluorometric TUNEL System (Promega, Madison, WI, USA). At least three samples were analyzed for each experimental group and time point.

### Global miRNA and mRNA Expression Profiling

A detailed global analysis of miRNA expression and its correlation with the transcriptome was performed using the miScript miRNA PCR array (Qiagen), GeneChip miRNA 4.0 array and GeneChip Mouse Transcriptome Array 1.0 (MTA 1.0) (Affymetrix, Santa Clara, CA, USA), covering 100% of miRBase v20 mouse miRNAs and 100% of the mouse transcriptome.

Three miScript miRNA PCR Arrays (Qiagen; MIMM-001Z) were used to analyze 12 samples from pooled retinas, corresponding to two samples per experimental group, rd10 and WT, from three time points: P13, P15, and P17. Each sample consisted of a pool of nine retinas from six mice.

Ten GeneChip miRNA 4.0 arrays and 10 GeneChip Mouse Transcriptome Arrays 1.0 were used to analyze 10 samples from pooled retinas: 3 for each rd10, from P13, P15, and P17 and 1 for WT from P13. Each sample consisted of a pool of nine retinas from six mice (Supplementary Fig. S1).

We first performed analysis of raw data from all GeneChip arrays, including a detection step (a probe set being detected above background with an associated *P* value) resulting in an absence/presence call and a quantile normalization step using the Expression Console Software v 1.4.1 (Affymetrix). In a subsequent filtering step, all nonmouse probe sets and those that were not detected (values below the background signal) in any of the arrays were removed to ensure that we worked with miRNAs and mRNAs actually expressed in mouse retina.

For identification of differentially expressed (DE) miRNAs and mRNAs, we used as reference rd10 and WT mice at P13 (in GeneChip arrays), 3 days prior to the onset of apoptosis. Adjusted *P* < 0.05 was considered statistically significant, and fold-change cutoffs of ±1.5 and ±1.3 were used for miRNAs and mRNAs, respectively. Further criteria used included the exclusion of miRNAs with values close to the background signal and triplicates with a high SD.

We then filtered the list of DE miRNAs based on fold-change values and on initial prediction studies of their putative target mRNAs (i.e., whether the corresponding genes were potentially relevant to retinal pathways in health and disease, such as genes known to be involved in apoptosis, inflammation, or normal retinal function or previously reported to be related to RP).

Candidate miRNAs were further classified into two groups based on their expression dynamics (i.e., whether altered expression occurred prior to or just after the onset of photoreceptor apoptosis) as follows: (1) group A, miRNAs with a significant up/downregulation at P13 and P15 compared with age-matched WT (P13 and P15) and rd10 at P13; and (2) group B, miRNAs with a differential expression at P17 compared with age-matched WT (P17) and with rd10 at P13: with no differential expression found at previous stages. In line with these criteria, we considered group A to contain the miRNAs that were most likely to be involved in the disease etiology and group B the miRNAs that were more likely to be involved in compensatory mechanisms and/or in disease progression.

### miRNA–mRNA Interaction Network Analysis and Gene Ontology and Pathway Enrichment Analysis

In silico prediction of mRNA targets for DE miRNAs was obtained using the miRWalk 2.0 database (available in the public domain, http://zmf.umm.uni-heidelberg.de/apps/zmf/mirwalk2/),^32^ which is able to predict targets for miRNAs using the algorithms of several databases including miRWalk 2.0 itself, miRanda,^33,34^ TargetScan,^35^ and RNA22,^36^ among others. Using Cytoscape v3.0.0 open access software,^37^ we constructed miRNA–mRNA interaction networks with both DE miRNAs and their target mRNAs as predicted by miRWalk 2.0. Only genes predicted by at least two algorithms were considered for further analysis.

To identify inverse expression relationships among miRNAs and mRNAs, we filtered these networks by incorporating the data from our mouse transcriptome arrays, selecting mRNAs with enriched expression in the retina (signal values above background) and mRNAs with an opposite fold change to that of their predicted regulator miRNAs. We generated three interaction networks, namely group A miRNAs versus DE mRNAs at P15 and at P17 and group B miRNAs versus DE mRNAs at P17.

**Figure 1:**
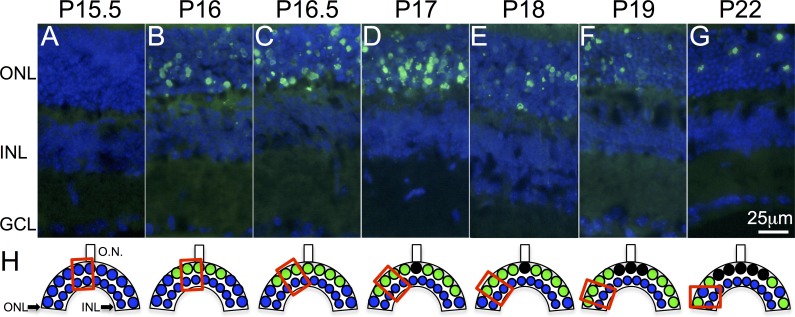
Photoreceptor cell death in rd10 mouse retina. (A, B) PR cell death (green nuclei) starts abruptly at P16 in the central retina, with no apoptotic nuclei detected neither at P13.5 (data not shown) nor at P15.5. (C–G) Cell death progresses toward the periphery. (H) Schematic representation of the PR cell death process. Preapoptotic nuclei are represented by blue circles and apoptotic nuclei are represented by green circles. Red squares represent the retinal areas where images were captured. Cell nuclei (blue) are labeled with 4′,6-diamino-2-fenilindol (DAPI), GCL, ganglion cell layer; INL, inner nuclear layer; ONL, outer nuclear layer; ON, optic nerve.

**Figure 2:**
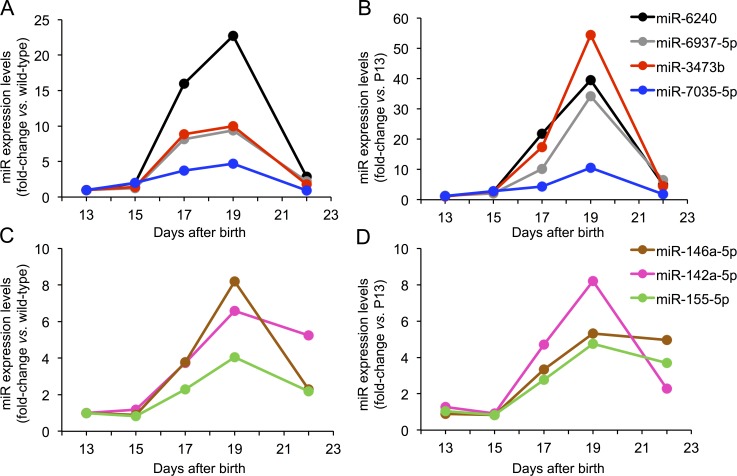
miRNAs expression levels in rd10 mouse retina. Differential miRNA expression at postnatal days 13, 15, 17, 19, and 22 are measured as the fold-changes compared with age-matched WT samples (left) and rd10 P13 samples (right). (A, B) Group A miRNAs. (C, D) Group B miRNAs. See Materials and Methods for a description of groups A and B.

**Figure 3:**
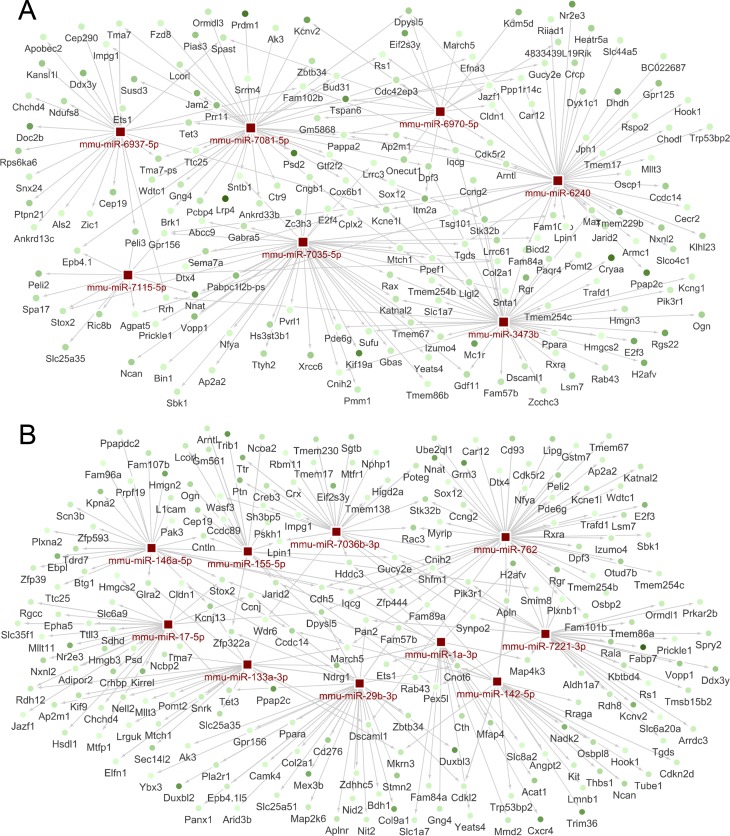
miRNA–mRNA interaction networks. A selection of representative differentially expressed miRNAs (red squares) and their predicted target genes with an inverse expression relationship (green circles). (A) miRNA–mRNA network of differentially expressed miRNAs prior to the onset of apoptosis (group A) and (B) just after the onset of apoptosis (group B). Arrows indicate direct interactions between an miRNA and its target genes. Changes in the shade of green of mRNA nodes reflect differences in fold-change, starting from 1.3-fold (light green) to up to −4.33-fold in group A and −7.65-fold in group B (dark green).

The differentially expressed putative target genes whose transcript levels were inversely expressed relative to the relevant DE miRNAs (inversely expressed genes) underwent analysis of gene ontology. For this purpose, we used CytoScape's ClueGO app (version 2.2.3) and analysis of biological pathways^38^ based on the Database for Annotation, Visualization and Integrated Discovery (DAVID, available in the public domain, http://david.abcc.ncifcrf.gov/) and WikiPathways (available in the public domain, www.wikipathways.org) to identify biological processes affected by miRNA dysregulation.

### Validation of miRNA and mRNA Expression by Quantitative PCR

Expression levels of DE miRNAs and inversely expressed mRNAs were analyzed for validation using a second technique, quantitative PCR (RT-qPCR). Validation was performed in the same samples as those studied in array analysis (i.e., three samples for each rd10 and WT mouse at three different time points [P13, P15, and P17]). RT-qPCR was conducted using miScript Primer Assays and the miScript SYBR Green PCR Kit (Qiagen, Hilden, Germany) for miRNA expression and the SYBR Green PCR Master Mix (Applied Biosystems, Foster City, CA, USA) for mRNA expression, according to the manufacturer's protocol. Each biological sample was amplified in triplicate. Threshold cycle values for miRNA expression were normalized to the mean expression values of miR-26a and miR-191-5p, which showed lowest variability of expression levels among all samples analyzed. For normalizing mRNA expression, we used the mean values of *Gapdh*, *Tubb5*, and β*-Act* genes for mRNA expression, which showed strong correlation between themselves in their levels of expression across all samples (*r*^2^ = 0.905 and *P* < 0.001).

## Results

Using the rd10 mouse model of RP, we conducted a detailed global study of the expression of more than 1900 miRNAs and the correlation of the levels of expression with the whole transcriptome to investigate possible differences in miRNA and mRNA expression profile between rd10 and WT mice retinas. This allowed us to identify a set of differentially expressed miRNAs and to analyze their possible role in the biological pathways involved in retinal degeneration through their predicted target genes.

### TUNEL Analysis

We carried out a detailed analysis of photoreceptor cell death dynamics in retinal sections obtained from mice ranging from P13 to P22 at 12-hour intervals to select the time points of interest for the miRNA–mRNA expression studies (Fig. 1). No TUNEL signal indicating apoptosis was detected prior to P16 (Figs. 1A–1F). A signal appeared on P16 in the central retina, mainly restricted to the outer nuclear layer (ONL) and progressed toward the peripheral retina, similar to what has been observed in previous studies.^28,29^ Based on our results, we selected retinal samples at three key time points for miRNA and mRNA expression profiling studies: prior to the onset of apoptosis at P13 and P15 and just after the onset of apoptosis at P17.

### miRNA and mRNA Expression Profiling

Based on the photoreceptor cell death dynamics, we analyzed the overall expression of miRNAs and mRNAs immediately prior to and after the onset of apoptosis, at P15, and at P17, when virtually all photoreceptors were still present. We also analyzed rd10 retinas at P13 to profile miRNA expression at an early stage, 3 days prior the onset of apoptosis. We considered that any possible alteration in the miRNA profile that could be happening in the rd10 mouse at this early stage is less likely to be influenced, if at all, by the apoptotic processes at this time.

The GeneChip miRNA 4.0 arrays revealed a large number of significantly differentially expressed (DE) miRNAs between the two strains with a greater than ±1.5-fold change (*P* < 0.05; Supplementary Table S1). Of more than 1900 miRNAs explored, 152 miRNAs were differentially expressed in the retina of rd10 mice with respect to control samples, and from this subset, we compiled a list of 19 candidate miRNAs for qRT-PCR validation analysis based on fold-change values and on preliminary prediction studies of their target mRNAs. In addition, miScript miRNA PCR arrays revealed a total of 18 DE miRNAs, this list being reduced to 6 candidate miRNAs by applying the same filtering criteria as for the GeneChip arrays (Supplementary Table S2). Therefore, our candidate miRNA list increased to 25 miRNAs, of which 19 were upregulated and 6 were downregulated.

Validation of candidate miRNAs was performed by qRT-PCR. Of 25 miRNAs tested, 19 miRNAs were validated. Data were presented as fold change over controls (Table 1). Most of the validated miRNAs were upregulated (17 of 19 miRNAs), and only two of six downregulated miRNAs were validated by qRT-PCR. Among validated miRNAs, the most highly upregulated were miR-6240 and miR-6970 (22-fold and 21-fold at P17, respectively), whereas miR-20b-5p and miR-19b-3p were the only miRNAs that were downregulated (−1.76-fold and −1.73-fold at P17, respectively).

**Table 1:**
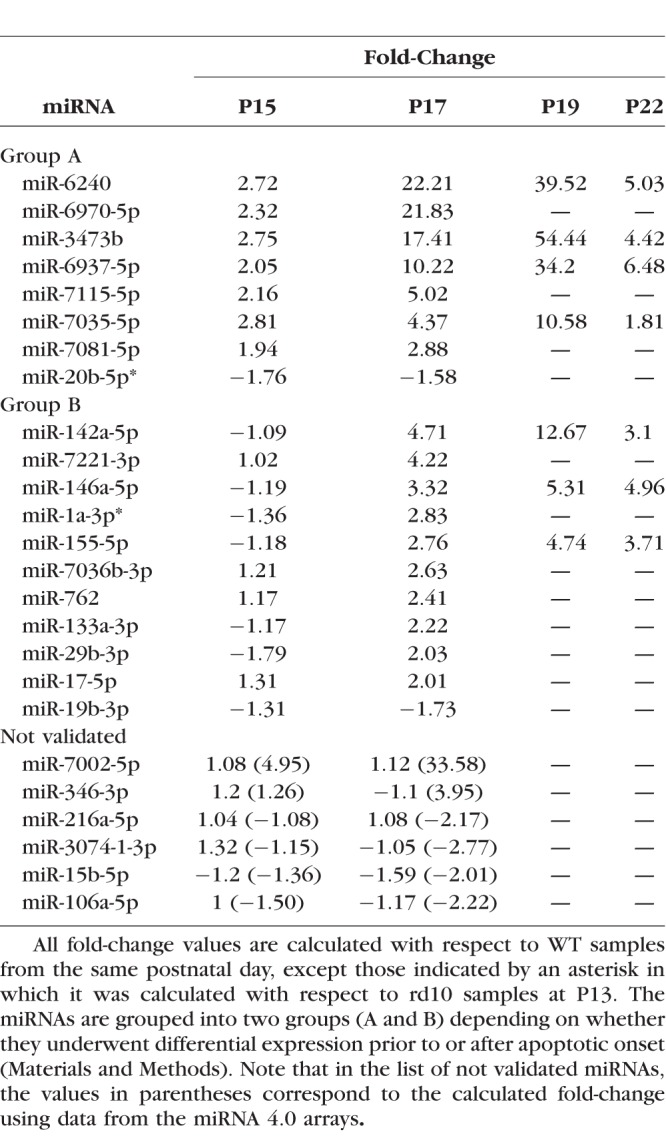
List of Differentially Expressed miRNAs Validated by RT-qPCR

The expression of three group B miRNAs, miR-155-5p, miR-142a-5p, and miR-146a-5p, were analyzed over a longer time frame, including measurements at P19 and P22. We selected these miRNAs based on the magnitude of the differential expression and/or the relevance of their predicted target genes in the disease. Expression of this group of miRNAs continued to increase at P19, reaching a plateau by P22, with expression values similar to those observed at P19 for miRs 155-5p and 146a-5p, and a marked drop in FC values from 12 to 3 for miR-142a-5p at P22 (Fig. 2). This pattern in expression levels from P19 might be indicating an exhaustion of compensatory mechanisms due to irreversible photoreceptor (PR) loss. Following the same selection criteria as that applied for group B, we selected four miRNAs to analyze over a longer time frame, namely, up to 22 days: miR-6240, miR-6937-5p, miR-3473b, and miR-7035-5p. The miRNA expression levels were still elevated at P19, decreasing to expression levels comparable to WT retinas by P22 (Fig. 2).

### Target Gene Analysis and miRNA–mRNA Networks

We identified an average of 900 target genes for each miRNA. Information from these target gene analyses was combined with data from miRNA expression profiling arrays to construct miRNA–mRNA regulatory networks using CytoScape open access software (Fig. 3; Supplementary Fig. S1). Then, we filtered these networks using data from our mouse transcriptome arrays. This approach allowed us to identify genes with levels of expression that were inversely correlated with those of our candidate miRNAs, which may play important roles in retinal degeneration. The 554 inversely expressed genes identified were analyzed using DAVID and WikiPathways open access databases to acquire knowledge about their biological relevance and to identify new actors and their involvement in the retinal degeneration process.

### Gene Ontology and Pathway Analysis

Target genes with an expression that was inversely correlated with that of the differentially expressed miRNAs subsequently underwent analysis of genetic ontology (GO) and biological pathways to improve our understanding of the mechanisms by which the candidate miRNAs might affect the rd10 mice phenotype. For this, we used DAVID and WikiPathways web services and CytoScape's ClueGO application. Ontology and biological pathway analysis revealed that a large number of inversely expressed targets are encoding components involved in biological functions likely to be involved in the pathogenesis of retinal degeneration, in compensatory mechanisms intended to prevent further retinal damage or in genes that are essential for retinal survival (Table 2). Considering these findings, we selected genes related to the pathways involved in apoptosis, inflammatory response, cytokine and chemokine activation, calcium signaling, visual perception, and visual phototransduction, among others, for qRT-PCR validation. In addition, we selected inversely expressed genes with high fold-change values, even if they had no clear involvement in retinal degeneration.

**Table 2:**
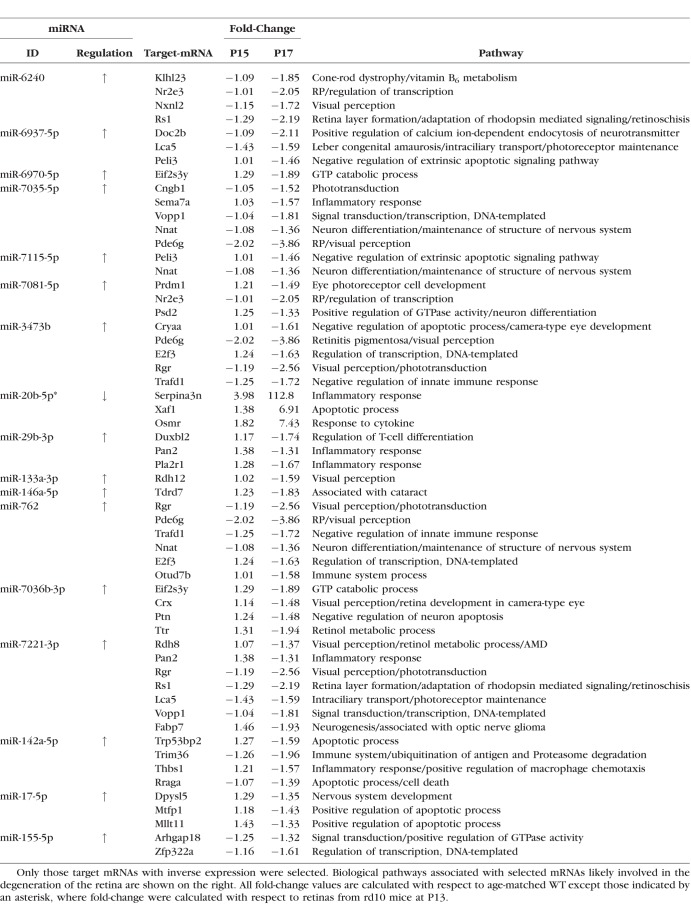
Summary of Differentially Expressed miRNAs and Their Predicted Target mRNAs

For target genes of the miRNAs that we classified into group A (i.e., differentially expressed prior to the onset of apoptosis), we mainly focused on pathways potentially involved in photoreceptor cell death such as apoptosis or inflammation, whereas for target genes of the miRNAs that we classified into the group B (i.e., differentially expressed just after the onset of apoptosis), we mainly focused on genes involved in pathways likely to be involved in photoreceptor survival. In addition to this, we also detected differentially expressed genes directly ascribed to retinal diseases such as *Klhl23* or *Nr2e3* involved in cone-rod dystrophies or RP, respectively. In total, we selected 49 genes for qRT-PCR validation.

Expression levels of these 49 genes, 45 downregulated and 4 upregulated, were analyzed by qRT-PCR for validation. Downregulation of 36 (80%) and upregulation of 3 (75%) of the selected genes was confirmed, summing to a total of 42 genes that showed differences in expression between rd10 and WT retinal samples. Table 2 summarizes information about the fold regulation of the candidate miRNAs, their inversely expressed target genes and their associated signaling pathways that might be involved in the retinal disease.

Finally, some of these genes have predicted interactions with more than one of the candidate miRNAs. For instance, *Pde6g*, which encodes the γ inhibitory subunit of the rod-specific phosphodiesterase 6, a key component of the phototransduction cascade, is a target for miR-3473b, miR-7035-5p, and miR-762. Another example is *Peli3*, a negative regulator of apoptosis, which is a predicted target for miR-6937-5p and miR-7115-5p.

## Discussion

In this work, we contributed to expanding our knowledge about the molecular mechanisms underlying RP. We identified a total of 19 candidate miRNAs that may play a fundamental role in the physiopathology and/or in the progression of the disease. GO and pathways enrichment analysis showed that some of the genes targeted by these miRNAs, which also showed differential expression between rd10 and WT mice, are involved in biological pathways with potential roles in retinal degeneration or survival.

Given the established role played by miRNAs in retinal development and survival,^1,2^ as well as in a growing number of distinct diseases,^7–10,12,14,15,19,22,39,40^ we hypothesized that alterations in miRNA expression might contribute to the molecular mechanisms involved in the physiopathology of RP. To provide experimental evidence to support the possible association between miRNA expression and retinal degeneration, miRNA expression profile in retinas from the rd10 RP mouse model^27–29^ and C57BL/6J wild-type mice were compared by microarray and RT-qPCR analysis in the early stages of retinal degeneration. To gain further insight into the possible relationship between miRNA alteration and gene expression regulation with implications in retinal degeneration, we also performed transcriptome expression profiling of the same samples as used for miRNA expression analysis.

We considered it likely that miRNAs down- or upregulated at a stage just prior to the onset of apoptosis might be associated with PR cell death mechanisms. Following the same rationale, we considered that miRNAs that are deregulated at latter stages might play relevant roles in compensatory mechanisms attempting to counteract PR death. Therefore, one of the key methodologic aspects of our study was to establish the time points for the miRNA–mRNA expression profiling analysis having first ascertained the exact time of onset of photoreceptor degeneration in our rd10 mouse model.

According to previous studies, the onset of PR apoptosis in the rd10 mouse was reported to take place between the second and third postnatal weeks.^27–29,41^ The onset of PR death is dependent on light conditions, with slight differences reported among different laboratories, depending on the intensity and duration of animals' exposure to light.^42^ To accurately establish the onset of the death of these cells, retinal samples were obtained every 12 hours between P13 and P22 and analyzed using TUNEL assay. We determined that apoptosis starts in the central retina between P15.5 and P16, with PR cell death then progressing toward the periphery (Figs. 1D–1F), in accordance with previous reports.^28,29,41^

One of the pathways that we focused on was inflammatory response; previous studies have evidenced that sustained chronic inflammatory process may contribute to the etiology of RP in rd10 mice and in other animal models of retinal degeneration.^43–46^ Furthermore, these studies support the notion that miRNAs are rapidly upregulated in response to inflammatory signals and may either stimulate the magnitude and duration of inflammation or silence it.^47^ Example of some of the genes related with inflammatory or immune response included Sema7a, Pan2, Pla2r1, Thbs1, or Trim36. Taking into account that deregulation calcium homeostasis plays a key role in retinal degeneration in rd10 mice, pathways involved the regulation, signaling, and/or exocytosis of this cation were also selected for validation, such as those related with Doc2b or Pde6g genes. In addition, we selected genes involved in physiologic processes or in the normal function of retinal cells, such as intraciliary transport (Lac5), phototransduction signal (Rgr), or retinol metabolism (Ttr).

Interestingly, among the inversely expressed genes, there were genes previously linked to retinal disorders. For instance *Klhl23* and *Nr2e3*, which are predicted targets of miR-6240, are linked with cone-rod dystrophy^48^ or RP,^49^ and *Rrg*, a predicted target of miR-3473b is associated with chorioretinal atrophy.^50^

With respect to most of the putative target genes, they showed a modest differential expression, in comparison with that found in the expression of their associated miRNA. That is, despite some miRNAs showing fold-change values greater than 5 or even 10, their predicted target genes showed a significant, although rather modest changes in their expression levels of about 1.5- to 2-fold, with the exception of *Serpina3n*, *Xaf1*, and *Osmr*, with fold-change values of 113, 7, and 7, respectively (Table 2). This difference in the magnitude of the expression levels of miRNAs and their target genes could be a consequence of the complex regulatory network in which a relatively few miRNAs can regulate as many as 50% to 60% of genes in humans and other mammals. Therefore, convergence of miRNA action on hundreds of genes implies that single miRNAs may have limited effect on the expression of their mRNA targets.^4^ Moreover, it should also be considered that some of the candidate miRNAs might be inhibiting protein translation rather than degrading mRNA. In such cases, mRNA levels would not be affected. Notwithstanding the fact that they showed significant but relatively small differences in expression, they are of potential interest in the context of molecular mechanisms underlying RP.

It is also remarkable that in most of genes with an inverse expression to that of group A miRNAs, that is, miRNAs deregulated at P15, showed significantly differential expression at P17, but not at P15. This delay might be related to the time that miRNAs require to exert their silencing effect on their mRNA targets. To the best of our knowledge, this is the first study that has described a time-delayed interaction between differentially expressed miRNAs and their predicted mRNA targets. This was possible through transcriptome analysis of retina samples at both P15 and P17, which allowed us to identify reverse expression relationships between DE miRNAs and their target mRNAs in a time resolved manner. In this regard, we considered that a 2-day lag time would allow a given miRNA to exert its inhibitory effect over its target mRNAs. Had we only studied miRNA and mRNA profiles at the same time points, the identification of this interaction would have been missed in most cases.

There are previous reports of the involvement of miRNAs in retinal development, function, and photoreceptors survival^1,2^ and also the involvement of different miRNAs in the pathogenesis of various diseases of the retina including miR-9, miR-34a, miR-125b, and miR-155 in macular degeneration,^17,18^ miR-146a and miR-195 in diabetic retinopathy,^19,20^ and miR-125a and miR-17 in retinoblastoma.^21,22^

Interestingly, some of the differentially expressed miRNAs we detected just after the onset of apoptosis are common to some of these retina-related miRNAs, including miR-146a, miR-155, miR-29b, and miR-17 (Table 1), as described below.

MiR-17 is a member of the miR-17-92 cluster that has been implicated in inflammatory diseases,^51^ Burkitt lymphoma,^52^ and retinoblastoma.^53^ Some evidence suggests that this cluster regulates a broad spectrum of biological processes of T-cell immunity including a negative expression relationship between miR-17 and the synthesis of Sirp-α gene that effectively regulate macrophage inflammatory responses.^54^

Among the retina-related miRNAs described above, we only found miR-17 and miR-29b to be inversely expressed with genes involved in pathways with a putative involvement in retinal degeneration. Examples of this are *Mtfp1* and *Mllt11*, both target genes for miR-17, and involved in positive regulation of apoptotic process.^55,56^ With regard to miR-29b, we detected downregulation of *Pla2r1*, *Pan2*, and *Duxbl2*, genes targeted by this miR that have been implicated in inflammatory and immune responses.^57^ Hence, increased expression of miR-17 and miR-29b could be part of survival mechanisms initiated to slow down the PR cell death process via the modulation of these genes.

As far as RP is concerned, Loscher et al. reported upregulation of miR-1a, miR-133a, and miR-142a in four different mouse models of RP linked to genes involved in both autosomal dominant and autosomal recessive forms of the disease, rhodopsin and rds/peripherin, respectively. Our results in rd10 mice are consistent with their findings.^25,26^ These results reinforce the hypothesis that there are commonalities in the miRNAs expression pattern in different types of RP regardless of the nature of their genetic cause, which could open the way to common therapeutic strategies applicable to different types of RP.

However, the role that upregulation of either miR-1 alone or the miR-1/133a cluster might be playing in the apoptotic process is not exempt from controversy. There are data suggestive of both anti- and proapoptotic effects on myocardiocytes under ischemic conditions, by targeting of the proapoptotic genes Casp9 and Bcl2^58,59^ and prosurvival genes including *Pkce*; *Hsp60*, and *Igf1*,^60,61^ respectively.

In any case, differential expression of miR-1a, miR-133a, and miR-142 in our study, as well as in the work of Loscher et al., is observed after the onset of apoptosis. This might be reflecting an activation of compensatory mechanisms in an attempt to prevent photoreceptor cell death in the mutant retina. In support of this, selective ablation of Müller cells resulted in photoreceptor death, and an altered expression of miR-1a, miR-133a, and miR-142.^39^ Hence, the observed increase in the expression of miR-142a and the miR-1a/133a cluster might be a direct consequence of the cell death process and a common feature in diseases that cause inflammation and apoptosis.

Among genes expressed inversely to miR-142a, we found genes involved in apoptosis (*Trp53bp2* and *Rraga*), and in inflammatory responses (*Thbs1* and *Trim36*). *Trp53bp2* acts as a positive regulator of the execution phase of apoptosis by encoding a member of the ASPP family (apoptosis-stimulating protein of p53) and *Rraga* plays a direct role in a *Tnf*α signaling pathway leading to induction of cell death.^62^ On the other hand, *Thbs1* is involved in inflammation through the activation of phospholipase C enzymes, which results in intracellular calcium levels rising and the activation of protein kinase C (PKC) in platelets,^63^ and *Trim36* is part of the superfamily of tripartite motif-containing (TRIM) proteins, which are induced by type I and II interferons and are crucial for induction of effective immunity.^64^ The fact that the expression of these genes decreased in the early stages of retinal degeneration may be indicating an attempt to counteract the cell death process by increasing the expression of miR-142a.

Differential expression of miR-7036b-3p, miR-7221-3p, and miR-762 was found at P17 compared with WT samples with no differential expression detected at P15. Among them, only one has previously been linked to disease, namely miR-762, which has been linked to vascular smooth muscle cell calcification^65^ and breast cancer.^66^ Notably, in the current study, we detected an inverse expression relationship between miR-762 and the *Pde6g* gene. As already discussed, *Pde6g* is a key player in the phototransduction cascade. Hence, increased levels of miR-762 might be contributing to exacerbate the death of rod cells in this mouse model with mutations in the Pde6b subunit by silencing the expression of the inhibitory γ subunit Pde6g of the PDE6 protein complex.

In addition, we found an inverse expression relationship of miR-762 with *Rgr*, *Trafd1*, *Nnat*, *E2f3*, and *Otud7b* mRNA, whose downregulation might be involved in the physiopathology of retinal degeneration, these genes having roles in relevant retinal processes such as phototransduction, negative regulation of the innate immune response, maintenance of nervous system structure, and regulation of transcription and immune system processes, respectively, according to GO and pathway enrichment analysis.

Interestingly, we found decreased expression of putative target genes for miR-7036b-3p and miR-7221-3p that might be involved in the development of retinal dystrophy rather than in compensatory mechanisms. Specifically, the target genes for miR-7036b include *Ptn*, a negative regulator of neuron apoptosis, and *Crx*, a causal gene of RP and other inherited retinal dystrophies. Further, one predicted target gene for 7221-3p is *Rdh8,* which encodes a retinol dehydrogenase that catalyzes the reduction of *all*-*trans*-retinal to *all-trans*-retinol, the first reaction step of the rhodopsin regeneration pathway, and its deficiency has been linked to AMD.^67^

Nevertheless, although it is possible that the above-mentioned genes, with significantly reduced levels of mRNA expression, are involved in the retinal degeneration in the rd10 mice, differential downregulation of miR-762, miR-7036b-3p, and miR-7221-3p takes place after the onset of PR apoptosis. Therefore, further studies are required to discern whether these three miRNAs are contributing to the physiopathology or to the progression of the retinal dystrophy.

Group B miRNAs, which have been previously associated with retinal diseases, were up- or downregulated after the onset of retinal degeneration. This might be indicating an involvement of these miRNAs in compensatory mechanisms against PR cells death or a deregulation resulting from PR cell death itself, and it is considered less likely that they contribute to disease onset.

In contrast, group A miRNAs showed differential expression at P15, prior to the onset of apoptosis. Therefore, this group of miRNAs may play important roles in the etiology of retinal dystrophy. Strikingly, to the best of our knowledge, none of the miRNAs included in group A have been previously linked to any retinal disease.

Group A is composed of seven miRNAs: miR-6937-5p, miR-6240, miR-3473b, miR-6970-5p, miR-7035-5p, miR-7081-5p, and miR-7115-5p. Notably, overexpression of all these miRNAs had increased by P17. For instance, miR-6937-5p showed a 2-fold upregulation at P15 and a 10-fold increase at P17.

Because most of the miRNAs we include in group A have only been described in recent years, there is little information regarding their possible involvement in different biological pathways. To the best of our knowledge, these miRNAs have not been ascribed to any disease. The expression of miR-6240-5p was more than 22-fold higher in rd10 retinas, this being the miRNA that showed the greatest upregulation. Among the target genes for miR-6240-5p, we found two genes associated with RP, *Klhl23* and *Nr2e3*, and one with retinoschisis, *Rs1*. Another noteworthy target for miR-6240 was Nxnl2, which has been suggested to be involved in the maintenance of both the function and the viability of sensory neurons, including photoreceptors.^68,69^

The second most upregulated miRNA was miR-6970-5p (Table 1). Inverse expression studies revealed *Eif2s3y*, *Onecut1*, and *Tspan6* genes to be negatively expressed with this miRNA. *Eif2s3y* participates in the GTP catabolic process^70^; *Onecut1* in transcription and cell cycle regulation^71^; and *Tspan6* mediates immune signaling in a ubiquitination-dependent manner.^72^

miR-6937-5p showed a significant 10-fold increase at P17. Its predicted targets, *Doc2b*, *Lca5,* and *Peli3*, were observed to be downregulated in our experiments. Doc2b is expressed ubiquitously and is suggested to be involved in calcium-dependent intracellular vesicle trafficking in various types of cells.^73,74^ Diseases associated with the *Lca5* gene include Leber congenital amaurosis and severe early-childhood-onset retinal dystrophy.^75,76^ This gene has functions in photoreceptor cell maintenance and in intraciliary protein transport as part of the centriole.^77^ With regard to *Peli3*, this gene acts as a negative regulator of the extrinsic apoptotic signaling pathway, and its suppression leads to enhanced formation of the death-induced signaling complex in response to TNF.^78^

Two interesting putative target genes for miR-3473b that we found to be downregulated were *Rgr* and *Pde6g*,^79–81^ both involved in the phototransduction signaling cascade: *Rgr* as part of the G-protein–coupled receptor signaling pathway and *Pde6g* encoding the gamma subunit of cyclic GMP-phosphodiesterase. *Pde6g* expression is restricted to PR, whereas *Rgr* is expressed in PR, RPE, and Müller cells.

Taking into account all the aforementioned findings regarding group A miRNAs, including the observed differential expression prior to the onset of apoptosis, the relevant role of their target genes for the retina in health and disease, and the sustained increased expression until at least P19, our data support the view that it is likely that this group of miRNAs plays an important role in the etiology of retinal dystrophy.

The observed downregulation/suppression of genes involved in apoptosis inhibition and inflammation regulation is in good agreement with the apoptotic death of PR cells and inflammatory processes observed in rd10 retinas, emphasizing the role that differentially expressed prior to the onset of apoptosis miRNAs might be playing in the retinal etiology.

Although it was initially proposed that miRNAs act mainly at the protein translation level, it is now widely accepted that both mRNA degradation and translation repression occur. However, despite great progress in elucidating the mechanism of miRNA-mediated mRNA targeting has been made, the extent and frequency of these two mechanisms has not yet been determined.^82,83^ In this regard, one of the limitations of our study is that it is focused only on the analysis of mRNA expression level.

Another limitation has to do with the predictive nature of our study. Despite many sequence-based target computational prediction algorithms having been developed and widely used to predict miRNA targets, their use implies inherent error in target predictions.^84^ These computational tools rely not only on the sequence complementary, but on thermodynamics, energy potentials, accessibility of target sites, and conservation, essentially scanning the entire transcriptome to predict a set of targets for each miRNA.^85^ However, this has the limitation of not considering that the relationship between miRNA and target genes is dynamic. This means that under different conditions, the same miRNA could have different sets of targets, and therefore different functions, when expressed in different cell types. Thus, these methods produce many false positives, even if the target information is accurate.^84^ To control false positives, a strict cutoff value is typically established; however, in this case, these methods tend to reject many true target relationships, that is, false negatives. Therefore, in this target prediction study, it is necessary to bear in mind that there is an inherent error that could condition our posterior results and that the inverse expression of the target mRNA of a particular miRNA is not at all proof of miRNA–mRNA regulation. Thus, miRNA–mRNA target pairs should be further validated in biological experiments, and therefore what our study is providing are strong candidates, which need further experimental validation.

## Conclusions

In the present work, we identified 19 DE miRNAs and 39 inversely expressed target mRNAs that may be key players during retinal degeneration, considering the reported involvement of these DE miRNAs and mRNAs in basic retinal processes such as apoptosis and inflammation regulation, which take place in degenerating rd10 retinas.

Among the miRNAs identified, seven have previously been reported in retinal studies, whereas nine have not previously been ascribed to any diseases.

As far as we are aware of, this is the first study describing a time-delayed interaction between differentially expressed miRNAs and their predicted mRNA targets. The identification of disease-related miRNAs and the consequent alteration in gene expression will contribute toward our understanding of the etiology and evolution of retinal degeneration.

## Supplementary Material

Supplement 1

## References

[1] Damiani D, Alexander JJ, O'Rourke JR, Dicer inactivation leads to progressive functional and structural degeneration of the mouse retina. *J Neurosci*. 2008; 28: 4878– 4887. 18463241 10.1523/JNEUROSCI.0828-08.2008 PMC3325486

[2] Sundermeier TR, Zhang N, Vinberg F, DICER1 is essential for survival of postmitotic rod photoreceptor cells in mice. *FASEB J*. 2014; 28: 3780– 3791. 24812086 10.1096/fj.14-254292 PMC4101655

[3] Pasquinelli AE. MicroRNAs and their targets: recognition, regulation and an emerging reciprocal relationship. *Nat Rev Genet* 2012; 13: 271– 282. 22411466 10.1038/nrg3162

[4] Friedman RC, Farh KK, Burge CB, Bartel DP. Most mammalian mRNAs are conserved targets of microRNAs. *Genome Res*. 2009; 19: 92– 105. 18955434 10.1101/gr.082701.108 PMC2612969

[5] Kim VN. MicroRNA biogenesis: coordinated cropping and dicing. *Nat Rev Mol Cell Biol*. 2015; 6: 376– 385. 10.1038/nrm1644 15852042

[6] Kjaer-Frifeldt S, Hansen TF, Nielsen BS, The prognostic importance of miR-21 in stage II colon cancer: a population-based study. *Br J Cancer*. 2012; 107: 1169– 1174. 23011541 10.1038/bjc.2012.365 PMC3461159

[7] Bertoli G, Cava C, Castiglioni I. MicroRNAs as biomarkers for diagnosis, prognosis and theranostics in prostate cancer. *Int J Mol Sci*. 2016; 17: 421. 27011184 10.3390/ijms17030421 PMC4813272

[8] Cui Z, Zheng X, Kong D. Decreased miR-198 expression and its prognostic significance in human gastric cancer. *World J Surg Oncol*. 2016; 14: 33. 26852230 10.1186/s12957-016-0784-x PMC4744396

[9] Xu P, Zhu Y, Sun B, Xiao Z. Colorectal cancer characterization and therapeutic target prediction based on microRNA expression profile. *Sci Rep*. 2016; 6: 20616. 26852921 10.1038/srep20616 PMC4745004

[10] van Rooij E, Marshall WS, Olson EN. Toward microRNA-based therapeutics for heart disease: the sense in antisense. *Circ Res*. 2008; 103: 919– 928. 18948630 10.1161/CIRCRESAHA.108.183426 PMC2725407

[11] Wang L, Yuan Y, Li J, MicroRNA-1 aggravates cardiac oxidative stress by post-transcriptional modification of the antioxidant network. *Cell Stress Chaperones*. 2015; 20: 411– 420. 25583113 10.1007/s12192-014-0565-9 PMC4406930

[12] Ghanbari M, Darweesh SK, de Looper HW, Genetic variants in MicroRNAs and their binding sites are associated with the risk of Parkinson disease. *Hum Mutat*. 2016; 37: 292– 300. 26670097 10.1002/humu.22943

[13] Munoz-Culla M, Irizar H, Saenz-Cuesta M, SncRNA (microRNA &snoRNA) opposite expression pattern found in multiple sclerosis relapse and remission is sex dependent. *Sci Rep*. 2016; 6: 20126. 26831009 10.1038/srep20126 PMC4735588

[14] Bhattacharyya S, Balakathiresan NS, Dalgard C, Elevated miR-155 promotes inflammation in cystic fibrosis by driving hyperexpression of interleukin-8. *J Biol Chem*. 2011; 286: 11604– 11615. 21282106 10.1074/jbc.M110.198390 PMC3064214

[15] Kumar P, Bhattacharyya S, Peters KW, miR-16 rescues F508del-CFTR function in native cystic fibrosis epithelial cells. *Gene Ther*. 2015; 22: 908– 916. 26133785 10.1038/gt.2015.56 PMC5488273

[16] Creemers EE, van Rooij E. Function and therapeutic potential of noncoding RNAs in cardiac fibrosis. *Circ Res*. 2016; 118: 108– 118. 26538569 10.1161/CIRCRESAHA.115.305242

[17] Karali M, Peluso I, Gennarino VA, miRNeye: a microRNA expression atlas of the mouse eye. *BMC Genomics*. 2010; 11: 715. 21171988 10.1186/1471-2164-11-715 PMC3018480

[18] Lukiw WJ, Surjyadipta B, Dua P, Common micro RNAs (miRNAs) target complement factor H (CFH) regulation in Alzheimer's disease (AD) and in age-related macular degeneration (AMD). *Int J Biochem Mol Biol*. 2012; 3: 105– 116. 22509485 PMC3325769

[19] Bhattacharjee S, Zhao Y, Dua P, microRNA-34a-mediated down-regulation of the microglial-enriched triggering receptor and phagocytosis-sensor TREM2 in age-related macular degeneration. *PLoS One*. 2016; 11: e0150211. 26949937 10.1371/journal.pone.0150211 PMC4780721

[20] Mortuza R, Feng B, Chakrabarti S. miR-195 regulates SIRT1-mediated changes in diabetic retinopathy. *Diabetologia*. 2014; 57: 1037– 1046. 24570140 10.1007/s00125-014-3197-9

[21] Kaidonis G, Gillies MC, Abhary S, A single-nucleotide polymorphism in the MicroRNA-146a gene is associated with diabetic nephropathy and sight-threatening diabetic retinopathy in Caucasian patients. *Acta Diabetol*. 2016; 53: 643– 650. 26997512 10.1007/s00592-016-0850-4

[22] Bai S, Tian B, Li A, MicroRNA-125b promotes tumor growth and suppresses apoptosis by targeting DRAM2 in retinoblastoma. *Eye (Lond)*. 2016; 12: 1630– 1638. 10.1038/eye.2016.189 PMC5177757 27518550

[23] Wang J, Wang X, Wu G, MiR-365b-3p, down-regulated in retinoblastoma, regulates cell cycle progression and apoptosis of human retinoblastoma cells by targeting PAX6. *FEBS Lett*. 2013; 587: 1779– 1786. 23660406 10.1016/j.febslet.2013.04.029

[24] Genini S, Guziewicz KE, Beltran WA, Altered miRNA expression in canine retinas during normal development and in models of retinal degeneration. *BMC Genomics*. 2014; 15: 172. 24581223 10.1186/1471-2164-15-172 PMC4029133

[25] Loscher CJ, Hokamp K, Kenna PF, Altered retinal microRNA expression profile in a mouse model of retinitis pigmentosa. *Genome Biol*. 2007; 8: R248. 18034880 10.1186/gb-2007-8-11-r248 PMC2258196

[26] Loscher CJ, Hokamp K, Wilson JH, A common microRNA signature in mouse models of retinal degeneration. *Exp Eye Res*. 2008; 87: 529– 534. 18834879 10.1016/j.exer.2008.08.016 PMC4030402

[27] Chang B, Hawes NL, Pardue MT, Two mouse retinal degenerations caused by missense mutations in the beta-subunit of rod cGMP phosphodiesterase gene. *Vision Res*. 2007; 47: 624– 633. 17267005 10.1016/j.visres.2006.11.020 PMC2562796

[28] Gargini C, Terzibasi E, Mazzoni F, Retinal organization in the retinal degeneration 10 (rd10) mutant mouse: a morphological and ERG study. *J Comp Neurol*. 2007; 500: 222– 238. 17111372 10.1002/cne.21144 PMC2590657

[29] Barhoum R, Martinez-Navarrete G, Corrochano S, Functional and structural modifications during retinal degeneration in the rd10 mouse. *Neuroscience*. 2008; 155: 698– 713. 18639614 10.1016/j.neuroscience.2008.06.042

[30] Hartong DT, Berson EL, Dryja TP. Retinitis pigmentosa. *Lancet*. 2006; 368: 1795– 1809. 17113430 10.1016/S0140-6736(06)69740-7

[31] McLaughlin ME, Ehrhart TL, Berson EL, Mutation spectrum of the gene encoding the beta subunit of rod phosphodiesterase among patients with autosomal recessive retinitis pigmentosa. *Proc Natl Acad Sci U S A*. 1995; 92: 3249– 3253. 7724547 10.1073/pnas.92.8.3249 PMC42143

[32] Dweep H, Gretz N. miRWalk2.0: a comprehensive atlas of microRNA-target interactions. *Nat Methods*. 2015; 12: 697. 26226356 10.1038/nmeth.3485

[33] Enright AJ, John B, Gaul U, MicroRNA targets in Drosophila. *Genome Biol*. 2003; 5: R1. 14709173 10.1186/gb-2003-5-1-r1 PMC395733

[34] John B, Enright AJ, Aravin A, Human MicroRNA targets. *PLoS Biol*. 2004; 2: e363. 15502875 10.1371/journal.pbio.0020363 PMC521178

[35] Lewis BP, Burge CB, Bartel DP. Conserved seed pairing, often flanked by adenosines, indicates that thousands of human genes are microRNA targets. *Cell*. 2005; 120: 15– 20. 15652477 10.1016/j.cell.2004.12.035

[36] Miranda KC, Huynh T, Tay Y, A pattern-based method for the identification of MicroRNA binding sites and their corresponding heteroduplexes. *Cell*. 2006; 126: 1203– 1217. 16990141 10.1016/j.cell.2006.07.031

[37] Smoot ME, Ono K, Ruscheinski J, Wang PL, Cytoscape 2.8: new features for data integration and network visualization. *Bioinformatics*. 2011; 27: 431– 432. 21149340 10.1093/bioinformatics/btq675 PMC3031041

[38] Bindea G, Mlecnik B, Hackl H, ClueGO: a Cytoscape plug-in to decipher functionally grouped gene ontology and pathway annotation networks. *Bioinformatics*. 2009; 25: 1091– 1093. 19237447 10.1093/bioinformatics/btp101 PMC2666812

[39] Chung SH, Gillies M, Sugiyama Y, Profiling of microRNAs involved in retinal degeneration caused by selective Müller cell ablation. *PLoS One*. 2015; 10: e0118949. 25741709 10.1371/journal.pone.0118949 PMC4351074

[40] Wang XJ, Huang B, Yang YM, Differential expression of microRNAs in aortic tissue and plasma in patients with acute aortic dissection. *J Geriatr Cardiol*. 2015; 12: 655– 661. 26788043 10.11909/j.issn.1671-5411.2015.06.013 PMC4712372

[41] Jae SA, Ahn KN, Kim JY, Electrophysiological and histologic evaluation of the time course of retinal degeneration in the rd10 mouse model of retinitis pigmentosa. *Korean J Physiol Pharmacol*. 2013; 17: 229– 235. 23776400 10.4196/kjpp.2013.17.3.229 PMC3682084

[42] Cronin T, Lyubarsky A, Bennett J. Dark-rearing the rd10 mouse: implications for therapy. *Adv Exp Med Biol*. 2012; 723: 129– 136. 22183325 10.1007/978-1-4614-0631-0_18 PMC4167785

[43] Liu Y, Yang X, Utheim TP, Correlation of cytokine levels and microglial cell infiltration during retinal degeneration in RCS rats. *PLoS One*. 2013; 8: e82061. 24349184 10.1371/journal.pone.0082061 PMC3862575

[44] Noailles A, Maneu V, Campello L, Persistent inflammatory state after photoreceptor loss in an animal model of retinal degeneration. *Sci Rep*. 2016; 6: 33356. 27624537 10.1038/srep33356 PMC5022039

[45] Smith JA, Das A, Ray SK, Role of pro-inflammatory cytokines released from microglia in neurodegenerative diseases. *Brain Res Bull*. 2012; 87: 10– 20. 22024597 10.1016/j.brainresbull.2011.10.004 PMC9827422

[46] Yoshida N, Ikeda Y, Notomi S, Laboratory evidence of sustained chronic inflammatory reaction in retinitis pigmentosa. *Ophthalmology*. 2013; 120: e5– e12. 10.1016/j.ophtha.2012.07.008 22986110

[47] Saxena K, Rutar MV, Provis JM, Identification of miRNAs in a model of retinal degenerations. *Invest Ophthalmol Vis Sci*. 2015; 56: 1820– 1829. 25711632 10.1167/iovs.14-15449

[48] Manes G, Hebrard M, Bocquet B, A novel locus (CORD12) for autosomal dominant cone-rod dystrophy on chromosome 2q24.2-2q33.1. *BMC Med Genet*. 2011; 12: 54. 21496248 10.1186/1471-2350-12-54 PMC3102607

[49] Escher P, Gouras P, Roduit R, Mutations in NR2E3 can cause dominant or recessive retinal degenerations in the same family. *Hum Mutat* 2009; 30: 342– 351. 19006237 10.1002/humu.20858 PMC3658139

[50] Bernal S, Calaf M, Garcia-Hoyos M, Study of the involvement of the RGR, CRPB1, and CRB1 genes in the pathogenesis of autosomal recessive retinitis pigmentosa. *J Med Genet*. 2013; 40: e89. 10.1136/jmg.40.7.e89 PMC1735523 12843338

[51] Lindberg RL, Hoffmann F, Mehling M, Altered expression of miR-17-5p in CD4+ lymphocytes of relapsing-remitting multiple sclerosis patients. *Eur J Immunol*. 2010; 40: 888– 898. 20148420 10.1002/eji.200940032

[52] Robaina MC, Faccion RS, Mazzoccoli L, miR-17-92 cluster components analysis in Burkitt lymphoma: overexpression of miR-17 is associated with poor prognosis. *Ann Hematol*. 2016; 95: 881– 891. 27044389 10.1007/s00277-016-2653-7

[53] Conkrite K, Sundby M, Mukai S, miR-17∼92 cooperates with RB pathway mutations to promote retinoblastoma. *Genes Dev*. 2011; 25: 1734– 1745. 21816922 10.1101/gad.17027411 PMC3165937

[54] Zhu D, Pan C, Li L, MicroRNA-17/20a/106a modulate macrophage inflammatory responses through targeting signal-regulatory protein alpha. *J Allergy Clin Immunol*. 2011; 132: 426– 436. 10.1016/j.jaci.2013.02.005 PMC5882493 23562609

[55] Tondera D, Santel A, Schwarzer R, Knockdown of MTP18, a novel phosphatidylinositol 3-kinase-dependent protein, affects mitochondrial morphology and induces apoptosis. *J Biol Chem*. 2004; 279: 31544– 31555. 15155745 10.1074/jbc.M404704200

[56] Co NN, Tsang WP, Tsang TY, AF1q enhancement of gamma irradiation-induced apoptosis by up-regulation of BAD expression via NF-kappaB in human squamous carcinoma A431 cells. *Oncol Rep*. 2010; 24: 547– 554. 20596645

[57] Granata F, Petraroli A, Boilard E, Activation of cytokine production by secreted phospholipase A2 in human lung macrophages expressing the M-type receptor. *J Immunol*. 2005; 174: 464– 474. 15611272 10.4049/jimmunol.174.1.464

[58] He B, Xiao J, Ren AJ, Role of miR-1 and miR-133a in myocardial ischemic postconditioning. *J Biomed Sci*. 2011; 18: 22. 21406115 10.1186/1423-0127-18-22 PMC3066105

[59] Tang Y, Zheng J, Sun Y, MicroRNA-1 regulates cardiomyocyte apoptosis by targeting Bcl-2. *Int Heart J*. 2009; 50: 377– 387. 19506341 10.1536/ihj.50.377

[60] Pan Z, Sun X, Ren J, miR-1 exacerbates cardiac ischemia-reperfusion injury in mouse models. *PLoS One*. 2012; 7: e50515. 23226300 10.1371/journal.pone.0050515 PMC3511560

[61] Yu XY, Song YH, Geng YJ, Glucose induces apoptosis of cardiomyocytes via microRNA-1 and IGF-1. *Biochem Biophys Res Commun*. 2008; 376: 548– 552. 18801338 10.1016/j.bbrc.2008.09.025

[62] Li Y, Kang J, Horwitz MS. Interaction of an adenovirus 14.7-kilodalton protein inhibitor of tumor necrosis factor alpha cytolysis with a new member of the GTPase superfamily of signal transducers. *J Virol*. 1997; 71: 1576– 1582. 8995684 10.1128/jvi.71.2.1576-1582.1997 PMC191215

[63] McLaughlin JN, Mazzoni MR, Cleator JH, Thrombin modulates the expression of a set of genes including thrombospondin-1 in human microvascular endothelial cells. *J Biol Chem*. 2005; 280: 22172– 22180. 15817447 10.1074/jbc.M500721200

[64] Ozato K, Shin DM, Chang TH, TRIM family proteins and their emerging roles in innate immunity. *Nat Rev Immunol*. 2008; 8: 849– 860. 18836477 10.1038/nri2413 PMC3433745

[65] Gui T, Zhou G, Sun Y, MicroRNAs that target Ca(2+) transporters are involved in vascular smooth muscle cell calcification. *Lab Invest*. 2012; 92: 1250– 1259. 22688076 10.1038/labinvest.2012.85

[66] Li Y, Huang R, Wang L, microRNA-762 promotes breast cancer cell proliferation and invasion by targeting IRF7 expression. *Cell Prolif*. 2015; 48: 643– 649. 26597380 10.1111/cpr.12223 PMC6496530

[67] Maeda T, Maeda A, Matosky M, Evaluation of potential therapies for a mouse model of human age-related macular degeneration caused by delayed all-trans-retinal clearance. *Invest Ophthalmol Vis Sci*. 2009; 50: 4917– 4925. 19494204 10.1167/iovs.09-3581 PMC2782428

[68] Elachouri G, Lee-Rivera I, Clerin E, Thioredoxin rod-derived cone viability factor protects against photooxidative retinal damage. *Free Radic Biol Med*. 2015; 81: 22– 29. 25596499 10.1016/j.freeradbiomed.2015.01.003

[69] Jaillard C, Mouret A, Niepon ML, Nxnl2 splicing results in dual functions in neuronal cell survival and maintenance of cell integrity. *Hum Mol Genet*. 2012; 21: 2298– 2311. 22343139 10.1093/hmg/dds050 PMC3664437

[70] Mazeyrat S, Saut N, Grigoriev V, A Y-encoded subunit of the translation initiation factor Eif2 is essential for mouse spermatogenesis. *Nat Genet*. 2011; 29: 49– 53. 10.1038/ng717 11528390

[71] Iyaguchi D, Yao M, Watanabe N, DNA recognition mechanism of the ONECUT homeodomain of transcription factor HNF-6. *Structure*. 2007; 15: 75– 83. 17223534 10.1016/j.str.2006.11.004

[72] Wang Y, Tong X, Omoregie ES, Tetraspanin 6 (TSPAN6) negatively regulates retinoic acid-inducible gene I-like receptor-mediated immune signaling in a ubiquitination-dependent manner. *J Biol Chem*. 2012; 287: 34626– 34634. 22908223 10.1074/jbc.M112.390401 PMC3464568

[73] Giladi M, Michaeli L, Almagor L, The C2B domain is the primary Ca2+ sensor in DOC2B: a structural and functional analysis. *J Mol Biol*. 2013; 425: 4629– 4641. 23994332 10.1016/j.jmb.2013.08.017

[74] Orita S, Sasaki T, Naito A, Doc2: a novel brain protein having two repeated C2-like domains. *Biochem Biophys Res Commun*. 1995; 206: 439– 448. 7826360 10.1006/bbrc.1995.1062

[75] Corton M, Avila-Fernandez A, Vallespin E, Involvement of LCA5 in Leber congenital amaurosis and retinitis pigmentosa in the Spanish population. *Ophthalmology*. 2014; 121: 399– 407. 24144451 10.1016/j.ophtha.2013.08.028

[76] Walia S, Fishman GA, Jacobson SG, Visual acuity in patients with Leber's congenital amaurosis and early childhood-onset retinitis pigmentosa. *Ophthalmology*. 2010; 117: 1190– 1198. 20079931 10.1016/j.ophtha.2009.09.056

[77] Gupta GD, Coyaud E, Goncalves J, A dynamic protein interaction landscape of the human centrosome-cilium interface. *Cell*. 2015; 163: 1484– 1499. 26638075 10.1016/j.cell.2015.10.065 PMC5089374

[78] Yang Z, Su D, Li Q, A R54L mutation of CRYAA associated with autosomal dominant nuclear cataracts in a Chinese family. *Curr Eye Res*. 2013; 38: 1221– 1228. 24074001 10.3109/02713683.2013.811260

[79] Dvir L, Srour G, Abu-Ras R, Autosomal-recessive early-onset retinitis pigmentosa caused by a mutation in PDE6G, the gene encoding the gamma subunit of rod cGMP phosphodiesterase. *Am J Hum Genet*. 2010; 87: 258– 264. 20655036 10.1016/j.ajhg.2010.06.016 PMC2917712

[80] Ksantini M, Senechal A, Bocquet B, Screening genes of the visual cycle RGR, RBP1 and RBP3 identifies rare sequence variations. *Ophthalmic Genet*. 2010; 31: 200– 204. 21067480 10.3109/13816810.2010.512354

[81] Trifunovic D, Karali M, Camposampiero D, A high-resolution RNA expression atlas of retinitis pigmentosa genes in human and mouse retinas. *Invest Ophthalmol Vis Sci*. 2008; 49: 2330– 2336. 18281612 10.1167/iovs.07-1513

[82] Catalanotto C, Cogoni C, Zardo G. MicroRNA in control of gene expression: an overview of nuclear functions. *Int J Mol Sci*. 2016; 17: 1712. 10.3390/ijms17101712 PMC5085744 27754357

[83] Djuranovic S, Nahvi A, Green R. miRNA-mediated gene silencing by translational repression followed by mRNA deadenylation and decay. *Science* 2012; 336: 237– 240. 22499947 10.1126/science.1215691 PMC3971879

[84] Oh M, Rhee S, Moon JH, Literature-based condition-specific miRNA-mRNA target prediction. *PLoS One*. 2017; 12: e0174999. 28362846 10.1371/journal.pone.0174999 PMC5376335

[85] Ovando-Vazquez C, Lepe-Soltero D, Abreu-Goodger C. Improving microRNA target prediction with gene expression profiles. *BMC Genomics*. 2016; 17: 364. 27189211 10.1186/s12864-016-2695-1 PMC4869178

