# Supplementary material for: Expression Profiling Analysis Reveals Key MicroRNA–mRNA Interactions in Early Retinal Degeneration in Retinitis Pigmentosa
Source: Invest Ophthalmol Vis Sci. 2018 May;59(6):2381–92. doi: 10.1167/iovs.18-24091 (PMC5939684; doi:10.1167/iovs.18-24091)
Supplement: Supplement 1 [file iovs-59-05-36_s01.pdf]

Supporting information

Expression profiling analysis reveals key microRNA-mRNA interactions in early retinal degeneration in retinitis pigmentosa.

Ander Anasagasti[1]; Maitane Ezquerra-Inchausti[1,2]; Olatz Barandika[1]; Maider Muñoz-Culla[3,4]; María M. Caffarel[5,6]; David Otaegui[3,4]; Adolfo López de Munain[1,7,8 9]; Javier Ruiz-Ederra*[1,2].

[1]Neuroscience Area, Sensorial Neurodegeneration Group, Biodonostia Health Research Institute, San Sebastian, Spain; [2]RETICS OFTARED, National Institute of Health Carlos III, Ministry of Economy and Competitiveness, Spain; [3]Neuroscience Area, Multiple Sclerosis Group, Biodonostia Health Research Institute, San Sebastian, Spain; [4]REEM: Spanish network on Multiple Sclerosis (Red Española de Esclerosis Múltiple); [5]Oncology Area, Biodonostia Health Research Institute, San Sebastian, Spain; [6]Ikerbasque, Basque Foundation for Science, Bilbao, Spain; [7]Department of Neurology, Donostia University Hospital, San Sebastian, Spain; [8]CIBERNED, Centro de Investigaciones Biomédicas en Red sobre Enfermedades Neurodegenerativas, Instituto Carlos III, Ministerio de Economía y Competitividad, Spain; [9]Department of Neuroscience, University of the Basque Country, San Sebastian, Spain
*Corresponding author.
E-mail: javiruizederra@yahoo.es/javier.ruizederra@osakideta.net (JR)

Supplementary Fig S1. **Workflow summarizing the approach used to identify candidate miRNAs and mRNAs possibly involved in retinal degeneration of rd10 mice.**

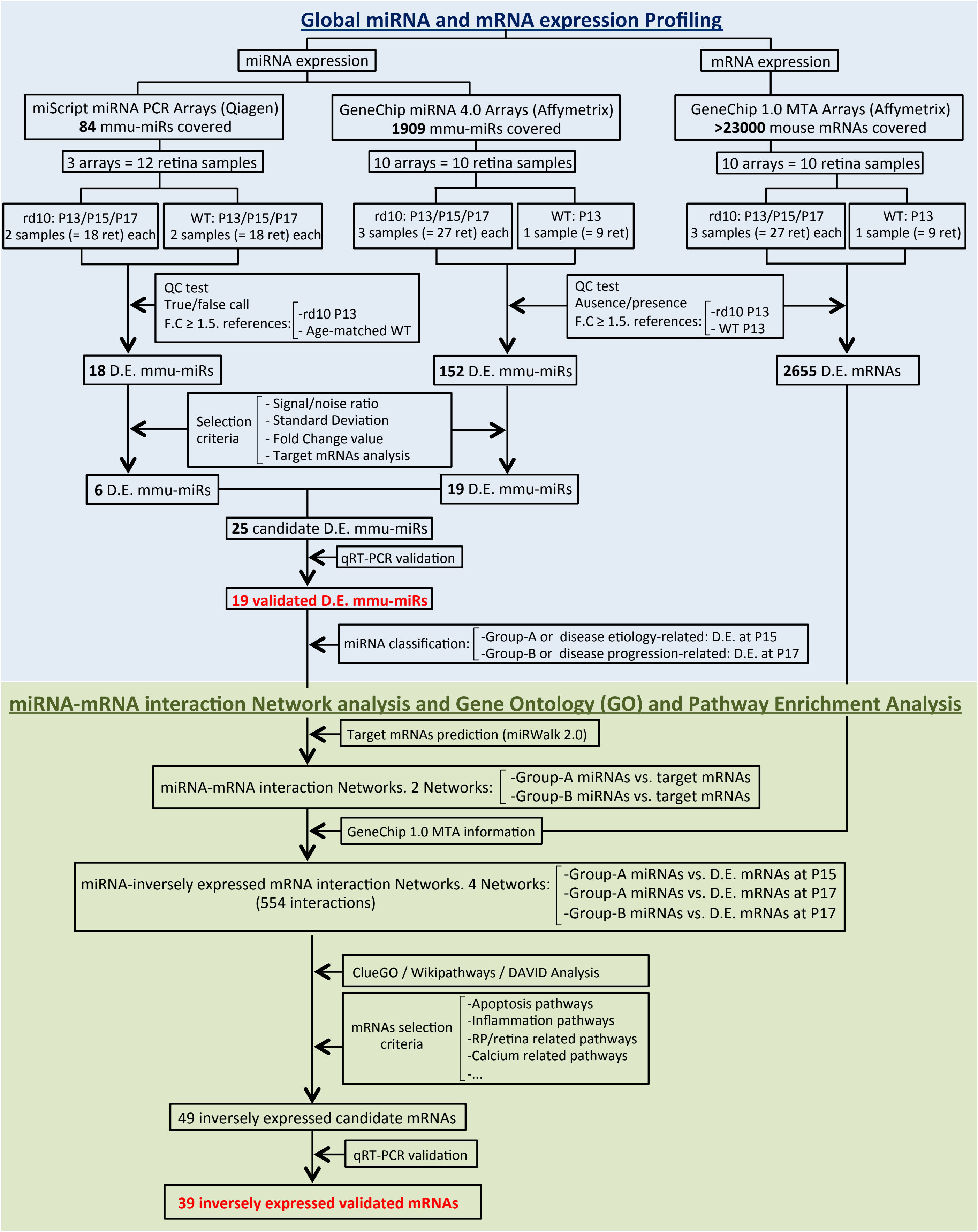

Supplementary Fig S1. Workflow summarizing the approach used to identify candidate miRNAs and mRNAs possibly involved in retinal degeneration of rd10 mice. Blue square summarizes global miRNA and mRNA expression profiling workflow to identify candidate miRNAs in which we used 2 miRNA profiling and one mRNA profiling systems. Green square summarizes miRNA-mRNA interaction network analysis generated with CytoScape software and Gene Ontology and Pathway Enrichment Analysis to identify mRNAs possibly involved in retinal degeneration of rd10 mice using ClueGO app and WikiPathways and DAVID databases. Abbreviations: DE differentially expressed; F.C.: fold-change; mmu-miRNA: *Mus musculus* microRNA; MTA: mouse transcriptome array; P13/15/17: postnatal days 13/15/17; qRT-PCR: quantitative reverse transcription polymerase chain reaction Q.C: quality control; SD: standard deviation.

Supplementary Table S1: Differentially expressed miRNAs. (F.C.> 1.5 and p value <0.05) detected by the GeneChip miRNA 4.0 arrays. In bold those values that meet these criteria.

Supplementary Table S2: Differentially expressed miRNAs (F.C.> 1.5) detected by the miScript miRNA PCR arrays. In bold those values that meet these criteria.

Supplementary Table S1: Differentially expressed miRNAs detected by the GeneChip miRNA 4.0 arrays.

Transcript ID	p15 vs. p13	p-value	p17 vs. p13	p-value
mmu-miR-7002-5p	4,95	0,0076	33,58	1,54E-07
mmu-miR-6937-5p	4,33	0,0031	20,40	0,0001
mmu-miR-7221-3p	1,68	0,1935	6,94	0,0021
mmu-miR-6970-5p	1,56	0,0099	6,47	0,0002
mmu-miR-6240	1,50	0,2283	6,21	0,0001
mmu-miR-7035-5p	1,16	0,8146	6,16	0,0183
mmu-miR-3473e	1,13	0,4942	5,73	0,0001
mmu-miR-3473b	1,16	0,3833	5,58	0,0001
mmu-miR-7036b-3p	1,14	0,4144	5,39	0,0002
mmu-miR-7648-3p	1,10	0,5639	4,63	1,11E-05
mmu-miR-1934-3p	1,49	0,2549	4,54	0,0041
mmu-miR-6968-5p	1,17	0,3451	4,43	3,39E-05
mmu-miR-7036-5p	1,42	0,1709	4,41	0,0010
mmu-miR-3620-5p	1,27	0,2966	4,41	0,0013
mmu-miR-7081-5p	1,32	0,1995	4,25	0,0004
mmu-miR-3090-5p	1,42	0,2509	4,18	0,0006
mmu-miR-3547-5p	1,21	0,2958	3,97	4,54E-05
mmu-miR-346-3p	1,26	0,3833	3,95	0,0014
mmu-miR-7115-5p	1,65	0,0298	3,84	0,0038
mmu-miR-714	-1,14	0,4126	3,78	0,0002
mmu-miR-3473a	1,15	0,4477	3,54	0,0001
mmu-miR-762	1,08	0,5752	3,48	0,0007
mmu-miR-7686-5p	1,13	0,7945	3,42	0,0459
mmu-miR-6910-5p	-1,14	0,5114	3,36	0,0006
mmu-miR-187-5p	3,06	0,0317	3,36	0,0226
mmu-miR-2137	-1,10	0,4606	3,29	0,0003
mmu-miR-7009-5p	1,57	0,3318	3,25	0,0436
mmu-mir-3473a	1,04	0,8052	3,12	0,0097
mmu-miR-7047-5p	1,27	0,0405	3,08	2,89E-05
mmu-miR-7658-5p	1,41	0,1639	3,03	0,0005
mmu-miR-5122	-1,05	0,8694	2,92	0,0085
mmu-miR-92a-2-5p	-1,05	0,8881	2,88	0,0007
mmu-miR-8102	-1,33	0,4832	2,67	0,0263
mmu-miR-5126	1,22	0,1290	2,66	0,0001
mmu-miR-5130	1,19	0,3204	2,62	0,0014
mmu-miR-3473f	1,47	0,2324	2,58	0,0156
mmu-miR-149-3p	1,17	0,3606	2,56	0,0001
mmu-miR-7023-5p	1,29	0,2788	2,55	0,0055
mmu-miR-6366	-1,18	0,3130	2,50	0,0020
mmu-miR-2861	1,22	0,2787	2,48	0,0015
mmu-miR-3102-5p	-1,08	0,7947	2,37	0,0089
mmu-miR-5128	1,10	0,6094	2,33	0,0050
mmu-miR-6944-5p	1,29	0,1685	2,32	0,0035
mmu-miR-3077-5p	1,17	0,3752	2,32	0,0022

mmu-miR-6909-5p	-1,07	0,5798	2,22	0,0029
mmu-miR-7069-5p	1,09	0,7143	2,19	0,0071
mmu-miR-6931-5p	1,23	0,2101	2,15	0,0052
mmu-miR-29a-3p	1,51	0,1741	2,11	0,0420
mmu-miR-6914-5p	1,48	0,0603	2,02	0,0098
mmu-mir-2861	1,10	0,2307	1,98	0,0003
mmu-miR-7085-5p	-1,12	0,6279	1,97	0,0036
mmu-miR-6394	1,18	0,2694	1,90	0,0120
mmu-miR-146a-5p	-1,14	0,5813	1,83	0,0387
mmu-miR-6956-5p	1,29	0,3086	1,83	0,0377
mmu-miR-150-3p	1,52	0,0415	1,81	0,0108
mmu-miR-6980-5p	1,04	0,8000	1,76	0,0396
mmu-miR-92b-5p	1,05	0,7156	1,76	0,0137
mmu-miR-6769b-5p	1,06	0,6857	1,70	0,0037
mmu-miR-3572-5p	1,11	0,2539	1,66	0,0140
mmu-miR-3960	1,14	0,2307	1,65	0,0027
mmu-miR-8101	1,00	0,9812	1,64	0,0038
mmu-miR-5132-5p	1,52	0,0119	1,60	0,0062
mmu-mir-3960	-1,10	0,1490	1,55	0,0006
mmu-mir-2137	-1,06	0,6307	1,51	0,0406
mmu-miR-3102-3p	1,58	0,0395	1,50	0,0544
mmu-miR-664-5p	1,91	0,0073	1,41	0,1123
mmu-miR-7682-3p	1,55	0,0099	1,37	0,0305
mmu-miR-193b-5p	1,77	0,0362	1,07	0,7644
mmu-miR-135a-1-3p	-1,86	0,0228	-1,42	0,0180
mmu-mir-25	-1,15	0,1076	-1,52	0,0116
mmu-miR-671-3p	1,17	0,2058	-1,53	0,0063
mmu-mir-677	-1,23	0,2669	-1,54	0,0125
mmu-miR-431-5p	-1,03	0,8297	-1,54	0,0136
mmu-miR-181c-5p	-1,18	0,2795	-1,54	0,0345
mmu-miR-199a-3p	-1,92	0,0501	-1,55	0,0346
mmu-miR-199b-3p	-1,92	0,0501	-1,55	0,0346
mmu-miR-181d-5p	-1,09	0,4286	-1,55	0,0038
mmu-miR-28a-3p	-1,28	0,0944	-1,55	0,0199
mmu-miR-494-3p	-1,20	0,1809	-1,56	0,0066
mmu-miR-9-3p	-1,26	0,1758	-1,57	0,0323
mmu-mir-6984	-1,19	0,2306	-1,58	0,0219
mmu-mir-183	-1,10	0,2229	-1,58	0,0015
mmu-miR-7a-2-3p	-1,11	0,3220	-1,59	0,0026
mmu-miR-669a-5p	-1,13	0,5404	-1,60	0,0327
mmu-miR-669p-5p	-1,13	0,5404	-1,60	0,0327
mmu-miR-505-5p	1,01	0,9363	-1,61	0,0097
mmu-miR-378a-3p	-1,03	0,7194	-1,62	0,0022
mmu-miR-423-3p	1,05	0,6603	-1,62	0,0214
mmu-miR-181a-2-3p	-1,21	0,0375	-1,64	0,0099
mmu-miR-532-3p	1,03	0,8243	-1,64	0,0108
mmu-miR-6965-5p	-1,05	0,7486	-1,64	0,0346
mmu-miR-672-5p	-1,20	0,0431	-1,65	0,0010
mmu-miR-378a-5p	1,03	0,6635	-1,65	0,0039

mmu-miR-17-3p	-1,04	0,7322	-1,65	0,0058
mmu-miR-217-5p	-1,16	0,1777	-1,66	0,0041
mmu-miR-6239	-1,33	0,1141	-1,67	0,0492
mmu-miR-676-3p	-1,16	0,0104	-1,68	0,0001
mmu-miR-183-3p	-1,16	0,1310	-1,68	0,0011
mmu-miR-5121	-1,21	0,0471	-1,68	0,0012
mmu-miR-34c-3p	-1,19	0,3408	-1,69	0,0248
mmu-miR-325-5p	-1,04	0,6819	-1,70	0,0002
mmu-miR-378c	-1,07	0,0664	-1,71	3,58E-05
mmu-miR-7a-1-3p	-1,38	0,1176	-1,71	0,0269
mmu-miR-484	-1,07	0,4473	-1,73	0,0063
mmu-miR-130b-3p	-1,13	0,1359	-1,73	0,0007
mmu-miR-30b-3p	-1,01	0,8906	-1,74	0,0008
mmu-miR-28a-5p	-1,15	0,1140	-1,75	0,0008
mmu-miR-378b	-1,11	0,1579	-1,75	0,0005
mmu-miR-378d	-1,09	0,1544	-1,81	0,0007
mmu-miR-124-5p	-1,12	0,3249	-1,81	0,0046
mmu-miR-181a-1-3p	-1,22	0,1793	-1,81	0,0461
mmu-mir-93	-1,11	0,2630	-1,81	0,0013
mmu-miR-30e-3p	-1,26	0,3502	-1,83	0,0195
mmu-miR-181c-3p	-1,09	0,5378	-1,84	0,0143
mmu-miR-6540-5p	-1,10	0,2450	-1,85	0,0016
mmu-miR-7007-5p	-1,10	0,5994	-1,86	0,0221
mmu-miR-3962	-1,68	0,0658	-1,88	0,0119
mmu-miR-467a-5p	-1,22	0,2160	-1,89	0,0048
mmu-miR-106b-3p	-1,10	0,3212	-1,91	0,0005
mmu-miR-34b-3p	-1,09	0,4136	-1,92	0,0014
mmu-miR-677-3p	-1,21	0,1801	-1,93	0,0045
mmu-miR-15a-5p	-1,21	0,4621	-1,96	0,0447
mmu-miR-350-3p	-1,37	0,1239	-1,96	0,0292
mmu-mir-1224	-1,12	0,4767	-1,97	0,0044
mmu-miR-3095-3p	-1,15	0,4495	-1,97	0,0381
mmu-miR-3078-5p	-1,22	0,0064	-2,00	2,29E-05
mmu-miR-15b-5p	-1,37	0,0533	-2,01	0,0018
mmu-miR-6984-5p	-1,15	0,4337	-2,02	0,0150
mmu-miR-106b-5p	-1,25	0,0551	-2,03	0,0001
mmu-miR-216b-5p	-1,46	0,0848	-2,04	0,0393
mmu-miR-351-5p	-1,36	0,0297	-2,05	0,0025
mmu-miR-503-5p	-3,06	0,0007	-2,07	0,0116
mmu-miR-92a-3p	-1,17	0,0382	-2,11	4,51E-06
mmu-miR-216a-5p	-1,09	0,4863	-2,16	0,0006
mmu-miR-17-5p	-1,38	0,0005	-2,17	2,48E-05
mmu-miR-296-3p	-1,16	0,2023	-2,18	0,0007
mmu-miR-93-3p	-1,29	0,1169	-2,19	0,0010
mmu-miR-20a-5p	-1,47	0,0006	-2,20	1,35E-05
mmu-miR-25-3p	-1,35	0,0791	-2,22	0,0044
mmu-miR-106a-5p	-1,50	0,0227	-2,22	0,0120
mmu-miR-449a-5p	-1,62	0,1199	-2,23	0,0353
mmu-miR-93-5p	-1,35	0,0253	-2,25	0,0004

mmu-miR-466a-3p	-1,65	0,1290	-2,38	0,0232
mmu-miR-466e-3p	-1,65	0,1290	-2,38	0,0232
mmu-miR-96-3p	-1,33	0,3054	-2,39	0,0262
mmu-miR-19b-3p	-1,48	0,0776	-2,51	0,0043
mmu-miR-20b-5p	-1,85	0,0151	-2,55	0,0018
mmu-miR-3074-1-3p	-1,15	0,5406	-2,81	0,0040
mmu-miR-18a-5p	-1,39	0,3300	-3,05	0,0145
mmu-miR-218-5p	-1,30	0,3910	-3,24	0,0103
mmu-miR-182-3p	-1,40	0,0596	-3,42	0,0019

Supplementary Table S2: Differentially expressed miRNAs detected by the miScript miRNA PCR arrays.

ID	p15	p17
mmu-miR-29a-3p	1,81	2,94
mmu-miR-146a-5p	1,15	2,5
mmu-miR-29b-3p	-1,14	1,91
mmu-miR-155-5p	1,19	1,77
mmu-miR-142a-5p	2,09	1,62
mmu-miR-1a-3p	-1,66	1,58
mmu-miR-22-3p	1,44	1,58
mmu-miR-17-5p	1,64	1,24
mmu-miR-96-5p	1,97	1,23
mmu-miR-101a-3p	1,68	1,2
mmu-miR-20a-5p	1,52	1,17
mmu-miR-18a-5p	2,01	-1,06
mmu-let-7b-5p	-1,66	-1,07
mmu-miR-19b-3p	1,67	-1,18
mmu-let-7c-5p	-1,85	-1,26
mmu-miR-183-5p	-1,29	-1,55
mmu-miR-92a-3p	1,24	-1,65
